# A little good is good enough: Ethical consumption, cheap excuses, and moral self-licensing

**DOI:** 10.1371/journal.pone.0227036

**Published:** 2020-01-15

**Authors:** Jannis Engel, Nora Szech

**Affiliations:** 1 Department of Economics, Karlsruhe Institute of Technology (KIT), Karlsruhe,Germany; 2 Berlin Social Science Center (WZB), Berlin, Germany; 3 CESifo, Munich, Germany; Shandong University of Science and Technology, CHINA

## Abstract

This paper explores the role of cheap excuses in product choice. If agents feel that they fulfill one ethical aspect, they may care less about other independent ethical facets within product choice. Choosing a product that fulfills one ethical aspect may then suffice for maintaining a high moral self-image in agents and render it easier to ignore other ethically relevant aspects they would otherwise care about more. The use of such cheap excuses could thus lead to a “*static moral self-licensing” effect*, and this would extend the logic of the well-known *dynamic moral self-licensing*. Our experimental study provides empirical evidence that the static counterpart of moral self-licensing exists. Furthermore, effects spill over to unrelated, ethically relevant contexts later in time. Thus, static moral self-licensing and dynamic moral self-licensing can exist next to each other. However, it is critical that agents do not feel that they fulfilled an ethical criterion out of sheer luck, that is, agents need some room so that they can attribute the ethical improvement at least partly to themselves. Outsiders, although monetarily incentivized for correct estimates, are completely oblivious to the effects of moral self-licensing, both static and dynamic.

## Introduction

The production of green, environmentally sustainable products has been increasing. This phenomenon includes products from organic agriculture [1, 2]and sustainably produced electronic items [3, 4]. However, even though consumers buy more green products, purchases seem to fall short compared with the stated intents that consumers express. Experts have argued that the ratio is one to ten: Spending on ethically improved products amounts to approximately 10% of what consumers claimed they would be willing to spend [5]. Moreover, data suggests that many consumers attempt to ignore ethically questionable production standards when possible [6]. This finding suggests that although many consumers have some awareness of ethical problems in production processes and some willingness to pay (WTP), cheap excuses may be welcome when regarding opting for less-ethical items.

Some brands, such as Wholefoods, foster a universal approach toward production that adheres to high, broad ethical standards. By contrast, many companies have focused on one ethical facet of their products while remaining rather silent on others. For example, fashion-producer H&M advertises that it replaces conventional cotton in its “conscious collection” with organic or recycled cotton [7]. In a similar manner, Apple publicly announced to audit of all its suppliers regarding the use of conflict minerals [8]. However, even though H&M and Apple have included the well-being of their workers in their codes of conduct [9–11], emphasizing its importance, reports of problematic working conditions have been repeatedly observed. For example, articles by Duhigg and Barboza [12], Burke [13]and Fullerton [14] have highlighted problematic working conditions in the production of Apple products, and Preston and Leffler [15] reported workers’ rights violations among some of the best-ranked suppliers of H&M.

We follow Bandura [16] and Gert [17] and define moral and ethical behavior as avoiding harm to other people and/or the environment. For a company, focusing on improving one ethical facet of their production process compared with many might be easier. For example, textile producers might limit environmental damage from growing cotton but ignore other ethically relevant aspects of their production processes. What might also be is that some facets such as environmental impact are easier to address than other facets such as labor and safety standards. This may partly explain why many companies stress specific ethical aspects to increase their ethical reputation. Another reason for such practices could be that they serve customers’ moral interests. Maybe, for customers, fulfilling one ethical aspect is sufficient to ease their moral conscience when buying a product. Potentially, for many product decisions, “a little good is good enough.” If so, the need to improve other ethical facets may become irrelevant for companies as soon as they address one aspect.

In three experiments, we explore whether subjects care comparatively less about another ethical, unrelated dimension as soon as one ethical aspect of a product appears to be fulfilled. For this purpose, in so-called Product treatments, we randomize subjects into different conditions and elicit their WTP for improved manufacturing standards of textile products. Subjects make decisions on towels in neutral colors and of a comparable size and weight (grammage of the cotton used). If they opt for a towel manufactured under certified conditions, they receive less money for themselves. Subjects make real economic decisions, that is, they receive a towel and money in the end, depending on their decisions in the study. Thus, in line with Levitt and List [18], we employ a real item to increase the outside validity of our economic experiments. We also elicit beliefs on how other subjects may behave in an incentivized manner. In all experiments, we follow the standards of economic experiments that have been described in Charness and Fehr [19] and Falk and Heckman [20].

In all treatments, the instructions inform subjects that manufacturing (i.e., sewing towels) is a production step unrelated to generating the raw material, in our case cotton, and that the two production steps often occur in different countries. Thus, whether a towel is made from conventional versus organic cotton has nothing to do with the manufacturing standards in the sewing step. Nevertheless, our data shows that the WTP for secure working standards in the sewing process is highly significantly smaller if subjects know that they decide between towels made from organic cotton instead of conventional cotton. The organic cotton is observed operate as a type of moral excuse to care less about the workers’ conditions in the sewing sector.

Next, we explore whether fulfilling one ethical dimension (organic cotton) also has spillovers to another, unrelated ethical context later in time and find the following. In our study, approximately half an hour after the main part of the experiment, in which the subjects’ focused on towels, they are offered the opportunity to share money with refugees from a local refugee camp. We find that subjects who know they will receive a towel made from organic cotton after the experiment donate less than subjects who know their towel will be made from conventional cotton. Again, organic cotton is observed to serve as an excuse to not care about another, ethically relevant context. We observe these effects even though subjects most likely knew that the refugee crisis was drastic when the experiments occurred, and that all help was needed to cope with it [21, 22].

Potentially, we find these static and dynamic moral spillovers because for the subjects, all ethical behaviors load on one and the same personality factor: “being an ethical person.” Akerlof and Kranton [23] argue that identity may play a critical role from which people derive positive utility. Falk and Szech [24] demonstrate that people care about their moral identity. However, maybe fulfilling one ethical aspect is sufficient for a decent moral identity and a positive self-image[25, 26].

Knowledge of these effects could be a critical first step to discussing social implications. Therefore, we incentivize new, independent subjects to predict the behavior of subjects in our original product choice treatments. Our data shows that these outsiders are completely unaware of the effects of static moral self-licensing, and they underestimate moral spillover effects in time. Potentially, outsiders follow a different moral compass and consider different product facets and contexts over time as what they are, namely, unrelated regarding consequences. Thus, outsiders do not anticipate that subjects, when weighing self-interest against something morally relevant, may prefer to find some type of moral excuse for self-oriented behavior.

The question arises as to whether self-attribution is critical for the small moral excuses we identify. Therefore, we run two additional treatments identical to the original product choice treatments, respectively, with one difference: The instructions stress and visually illustrate that it is perfectly random whether a subject makes decisions about towels made from conventional or from organic cotton. In this manner, organic cotton may become a less valid, self-attributable excuse to not care about sewing conditions and refugees, and this is indeed what we find.

Studies of economic institutions in which agents make morally relevant decisions are a pressing and strongly growing field within economics. Recent contributions from economics include Sobel [27], Falk and Szech [28], Kirchler et al. [29], and Pigors and Rockenbach [30] on market trading and morals. Falk and Szech [31] and Rothenhäusler et al. [32] have studied morals in group voting. Kerschbamer et al. [33], Bartling et al. [34], and Friedrichsen and Engelmann [35] have considered ethical consumption and/or morally relevant credence goods. Our results indicate that consumers’ moral excuses should receive specific attention in follow-up research.

## Literature review

Our results relate to the classic moral self-licensing effect. However, we explore a *static* decision context, whereas the classical literature on moral self-licensing has focused on *dynamic* effects only. Researchers have documented that ethical behavior in the past serves as a justification to act less ethically later. Such dynamic moral self-licensing was first described by Monin and Miller [36] in the contexts of racism and sexism in a two-stage experiment. Subjects had to make a hypothetical job decision in a neutrally framed environment. In one treatment group, the best applicant was African American, and in the other group, the best applicant was white. After making their decision, all subjects were confronted with another hypothetical hiring scenario: A chief of police had to hire a new deputy in a racially charged job environment. All subjects were asked to rate whether the job was better suited for a white or a black person. Subjects with the opportunity to present themselves as non-prejudiced before were significantly more likely to prefer a white person now. Thus, subjects who could demonstrate they were not racist in the first step, tended to prefer the white police applicant later. A second study by Monin and Miller [36] yielded similar findings in the field of sexism.

Sachdeva et al. [37] found that subjects use dynamic moral self-licensing to justify selfish behavior. Subjects asked to write positive short stories on their good past deeds were later observed to be more selfish than others regarding making a real donation to charity. In a related study, Mazar and Zhong [38] demonstrated that subjects who acquired “green” products in the first step are more likely to cheat for personal gain and steal later compared with subjects who could only acquire conventional products in the first step in time. For more examples of dynamic moral self-licensing, see Merritt et al. [39].

Another closely related concept from the field of behavioral economics is conscience accounting, proposed in Gneezy et al. [40]. In the first stage of their experiment, subjects could lie to increase their profit at the expense of their fellow players. In stage two, subjects had the opportunity to make a small donation to charity. The authors observed that the share of donations was significantly higher among the subjects who lied in stage one compared with those who told the truth. This finding suggests that by donating, subjects atone for past moral norm violations. Furthermore, the share of donations among the liars decreases significantly if the time delay between the two stages increases. A possible explanation for this finding is that the memory of an individual’s own unethical behavior fades over time. Another finding is that subjects are more likely to lie if they know that they have the opportunity to donate later. This leads to the conclusion that people set off their future ethical behavior against their current ethical behavior, and this conclusion is in line with the idea that all types of moral behavior may load on one aspect of personality if subjects can attribute those moral improvements to themselves [24, 25, 26]. The authors also documented that a comparatively cheap future possibility of “compensation” might be sufficient for subjects to maintain a high moral self-image.

What differentiates this approach from ours is that in our Product treatments, subjects make decisions on the static moral improvement (the upgrade to the fair wear towel at the dispense of different monetary amounts) in a price list. They do not know which of their decisions the computer will randomly select when they decide to donate money to refugees a half an hour later. Moreover, the subjects are unaware of the opportunity to make a donation beforehand. This design enables the subjects to focus on whether deciding about towels made from organic instead of conventional cotton is sufficient to care less about other ethically relevant aspects—static and dynamic.

## Design of the study

We explore the effects of moral self-licensing across different ethical facets in purchase decisions and across different points in time. To this end, we implement a product choice regarding different towels and a subsequent donation situation. In addition to behavior, we study beliefs of outsiders about the behavior of decision-makers to analyze whether beliefs of outsiders are in line with real choice behavior. Therefore, we conduct four treatments: **Product Control, Product Organic, Belief Control, and Belief Organic.**

In our experiment, we employed towels for the following three reasons. First, the value of the fashion industry is USD trillions, encompassing a wide variety of companies [41] and employs over 57 million people worldwide [42]. Thus, this sector is a critical industrial sector. Second, the bulk of the production occurs in developing and newly industrialized countries, such as China, India, Vietnam, Bangladesh [43, 44], and the textile industry has often been the focus of consumers’ demand for improved production standards. Third, we specifically used a product that we hoped most subjects found useful, independent of personal characteristics such as gender or taste in fashion. Therefore, the subjects made decisions about towels in neutral colors.

### Product treatments

In **Product Control** and in **Product Organic**, subjects made ethically relevant product decisions. Subjects were randomized into the two treatments. They choose between ethically different yet otherwise comparable towels. In a price list, they weighed money and receiving a more conventionally produced towel against receiving no money and a towel that fulfills a more ethical set of production standards. The subjects choose between towels in neutral colors; in a standard, medium size of 100 cm x 50 cm, and with a standard surface weight of approximately 450 g/m^2^.

### Belief treatments

In **Belief Control** and in **Belief Organic**, we elicited in an incentivized manner outsiders’ beliefs about the behavior of the subjects in Product Control and in Product Organic, respectively. The Belief treatments provided information on what the uninvolved third parties, who did not make the decisions, predicted about the individuals involved. Subjects were randomly assigned to the two Belief treatments. For organizational reasons, randomization in this study was at the session level.

In all treatments, the instructions informed subjects of the different ethically relevant dimensions in the production of textiles such as towels. We focused on two important, separate aspects. First, the cotton used could be conventional or certified “organic.” Here, we relied on the well-known Global Organic Textile Standard (GOTS) and the Organic Content Standard (OCS), which certify the organic origin of cotton. All subjects knew from the instructions that the certified organic cotton was produced without the use of agrochemicals, such as synthetic pesticides, herbicides, and fertilizers commonly used in conventional production and that pollute the soil. Second, the manufacturing conditions under which workers must work when sewing the towel can be certified by the well-established Fair Wear Foundation (FWF), or not. The FWF is a well-established, independent non-profit organization that aims to improve labor standards in factories based on the conventions of the International Labour Organization (ILO). Subjects were informed that under these FWF conditions, safety, economic living standards, and political rights of workers are monitored. These benefits are not guaranteed in conventional production. Indeed, on the topic of ethics, the textile and garment industry is one most frequently discussed industries, e.g., Danzer and Grundke [45].

The instructions also provided information on the fragmentation of production chain in the garment industry, that is, the cotton is typically grown and manufactured in different countries. Thus, subjects knew that these two aspects, growing organic cotton and ensuring FWF-controlled manufacturing standards, could be considered separate from each other.

To analyze moral self-licensing within product decisions, we elicited the monetary value subjects assigned to the FWF manufacturing standards in Product Control and in Product Organic. In Product Organic, subjects knew that the towel already fulfilled another independent, ethically relevant criterion (i.e., organic cotton). Decisions were elicited in a price list. Subjects choose between a towel that was manufactured under the FWF-controlled standards and no additional money versus a towel without FWF-controlled manufacturing standards and additional money. Monetary amounts varied from 0.25 to 12 euro in steps of 25 cents. Subjects knew that one of their decisions would be randomly selected at the individual level and implemented. That is, subjects received—in any case—a towel with the respective ethical attributes from their choice in the price list and, depending on the choice, additional money.

We defined the lowest monetary amount for which subjects still preferred no FWF certificate over the FWF certificate as the WTP for FWF certification. The lower the switch point, the less a subject cared about FWF-certified working standards. In cases of multiple switching, we used the mean switch point for our analysis. We observed that the results were robust to multiple switching.

**Fig 1:**
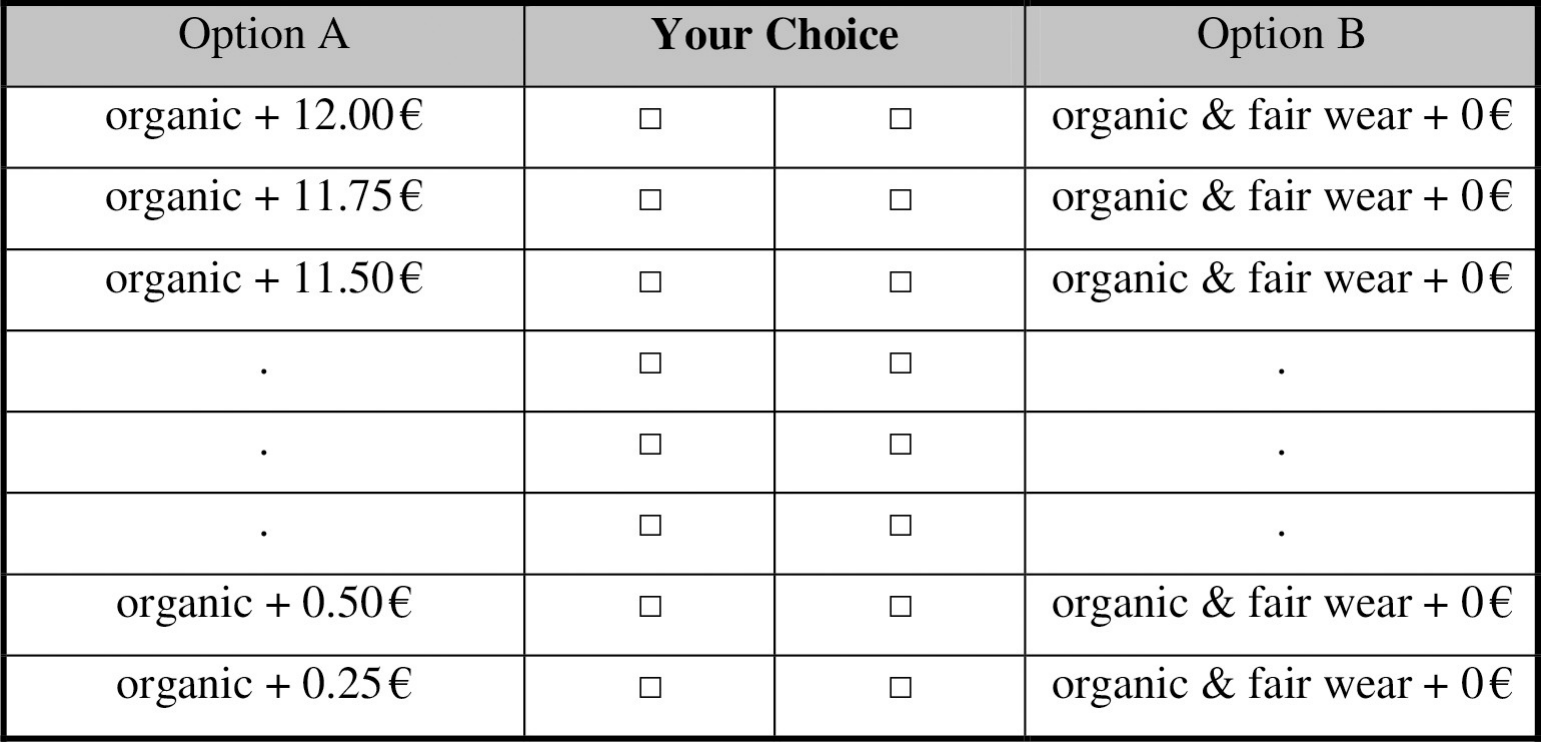
Price list as used in product organic. In the Organic Product treatment, subjects choose from a price list between a towel without an FWF certificate and money versus a towel with an FWF certificate and no additional money. The FWF certificate ensured controlled manufacturing standards for workers when sewing the towel. In any case, the towel is made from organic cotton.

Fig 1 illustrates the price list in Product Organic. Product Control was similar except that the cotton was conventional (see Appendix B in S1 Appendix. for details). After this main decision, subjects completed questionnaires on their political preferences, personal characteristics, and socioeconomic background. We used standard tests of personality, such as the Big Five [46], and of Machiavellianism [47] and the rather new Preference Survey Module [48]. At the end of the study, approximately half an hour later, the instructions confronted subjects with another ethically relevant decision. Subjects did not know this in advance, and therefore, compared with the study of Gneezy et al. [40], subjects could not use the later sharing decisions as a justification for selfish behavior in the towel decision. Through the computer screen, subjects had to decide whether they wanted to share their show-up fee (i.e., 2 euro) with refugees from a local refugee camp. We chose to implement a binary decision instead of a price list to distinguish this choice, also regarding its framing, from the previous decision on towels. The donation amount was substantial but not exceedingly high compared with the payout that subjects could earn in the study, which comprised the show-up fee, a towel, and a potential payoff from the price list. At this point, subjects had not received feedback on whether they would receive a towel produced under the FWF or conventional production standards.

In the **Belief treatments**, that is, in Belief Control and in Belief Organic, subjects did not make any ethically relevant product or donation decision themselves. Instead, subjects indicated their expectations of subjects in the respective Product treatments behaved. We incentivized these estimates through quadratic scoring rules. The respective estimation for the mean willingness to pay, given in cents, had the following payout: *max {500 - $\frac{1}{4}$ (true—guess)*^*2*^, *0}* cent.

In Belief Control, subjects guessed the average WTP for controlled manufacturing standards in Product Control. Subjects in Belief Control knew the instructions from the Product Control treatment for the towel decision. Thus, they had the exact same information as the subjects in Product Control. After indicating their estimate, they also filled out the same questionnaires as subjects in the Product treatments. Then, the instructions confronted them with a second estimation task, requiring them to guess what percentage of subjects in Control opted to share their show-up fee with refugees. Again, we employed a quadratic scoring rule to incentivize this task. The payout for the estimated willingness to share was *max {5 - $\frac{1}{5}$ (true–guess)*^*2*^, *0}* euro. Subjects had to type in integer percentage values.

In Belief Organic, subjects accordingly guessed the behavior from Product Organic. In total, subjects in Belief Control and in Belief Organic could earn up to 10 euro for good estimates, in addition to a show-up fee of 7 euro.

The study took place at the Karlsruhe Decision and Design Laboratory (KD^2^ Lab) at the Karlsruhe Institute of Technology. We used ORSEE [49] and hroot [50] to recruit 200 subjects (50 subjects per treatment). Subjects were placed in separate cubicles. Instructions regarding the towels were presented on paper, and questionnaires were presented on a computer screen by using SoSci Survey [51]. See Appendix B in S1 Appendix for details.

## Hypotheses

The insights gained from the experiments on moral self-licensing [36, 37, 38] and conscience accounting [40] show that people use acquired moral credentials to justify less-ethical behavior at a later point in time. In a similar fashion, they morally redeem themselves from unethical behavior in the past by performing subsequent ethical deeds. Even if the ethical deeds are small and cheap later on, they seem to serve as a good justification for rather selfish behavior at an earlier point in time [40]. Gneezy et al. [40] asserted that a similar logic may have been behind the sale of indulgences, as practiced by the Catholic Church in medieval times. Yet in that case, moral redemption was often costly.

Moral cleansing may provoke a reduction in moral behavior in the first place, specifically if subjects *know* about the possibility to morally cleanse themselves afterward [40]. Such a mechanism could also exist in the framework of a static decision across different ethically relevant facets of one product. The satisfaction of fulfilling one ethical dimension could lead to a decreased valuation of a second—though unrelated—ethical dimension at the same point in time. This would imply that the time dimension is not what renders moral cleansing relevant; instead, it is the opportunity to have some type of “moral excuse.” This logic would be specifically attractive if subjects did not care about consequences but instead considered all these decisions relevant for one specific aspect about themselves, that is, a positive self-image [24, 25, 26]. Our first and main hypothesis is therefore that subjects in Product Organic have a lower WTP for controlled manufacturing conditions than subjects in Product Control; thus, static moral self-licensing exists.

Therefore, we expect that

$${\mathrm{WTP}}_{\mathrm{Product}\;\mathrm{Organic}}<{\mathrm{WTP}}_{\mathrm{Product}\;\mathrm{Control}.}$$

In addition to the static product decision, subjects later had to make another unrelated ethical decision by choosing whether to share their show-up fee with local refugees. Because our subjects do not receive any feedback on their payoff from the price list until after the experiment, they know whether their towel is ethical in one dimension (the material) but cannot be sure whether it was produced under controlled manufacturing conditions. (The exception is subjects willing to pay the maximal amount or unwilling to pay the minimal amount for the controlled manufacturing conditions.) In line with the findings on dynamic moral self-licensing, the certainty of having acquired a product that fulfills (at least) one ethical facet in the first stage should “free” the subjects to behave less ethically afterward. This leads to our second hypothesis: Subjects in Product Organic will be less likely to share their show-up fee than subjects in Product Control, that is, static moral self-licensing and dynamic moral self-licensing can exist next to each other:

$${\mathrm{Donation}}_{\mathrm{Product}\;\mathrm{Organic}}<{\mathrm{Donation}}_{\mathrm{Product}\;\mathrm{Control}.}$$

Exploratively, we also investigate the incentivized beliefs of outsiders about the behavior of subjects in the respective Product treatments. Thus, we conduct two Belief treatments: Belief Control and Belief Organic. In these treatments, subjects do not face trade-offs between more ethical decisions and money. Instead, they are incentivized to predict the decisions of other subjects from the respective Product treatment.

It may be that subjects in the Belief treatments predict that moral self-licensing will take place in the Product treatments. This is not so clear, however, for several reasons. First, subjects in the Belief treatments do not face a trade-off between more ethical decisions and money. In the literature, hypothetical questions and real behavior have often correlated regarding altruistic versus selfish behavior. However, typically, in real behavior, subjects care much more about their self-interest than when hypothetically asked. Accordingly, the literature on ethical consumption speaks of the famous 30:3 ratio [5] or attitude behavior gap[52, 53], comparing stated intent from questionnaires to real decisions in the market. Furthermore, the literature has shown that context can affect morally relevant judgments (see e.g.,[16, 28, 29, 31, 34, 54, 55, 56]).

Therefore, we expect that

$${\mathrm{Belief}}_{\mathrm{WTP}\_\mathrm{Product}\;\mathrm{Organic}}\leq {\mathrm{Belief}}_{\mathrm{WTP}\_\mathrm{Product}\;\mathrm{Control},}$$

and accordingly

$${\mathrm{Belief}}_{\mathrm{Donation}\_\mathrm{Product}\;\mathrm{Organic}}\leq {\mathrm{Belief}}_{\mathrm{Donation}\_\mathrm{Product}\;\mathrm{Control}.}$$

If subjects do not predict that moral self-licensing will take place, that is, if both formulas hold with equality, emphasis on the importance of moral self-licensing in social and public debate might be relevant.

## Results

### Static moral self-licensing

**Fig 2:**
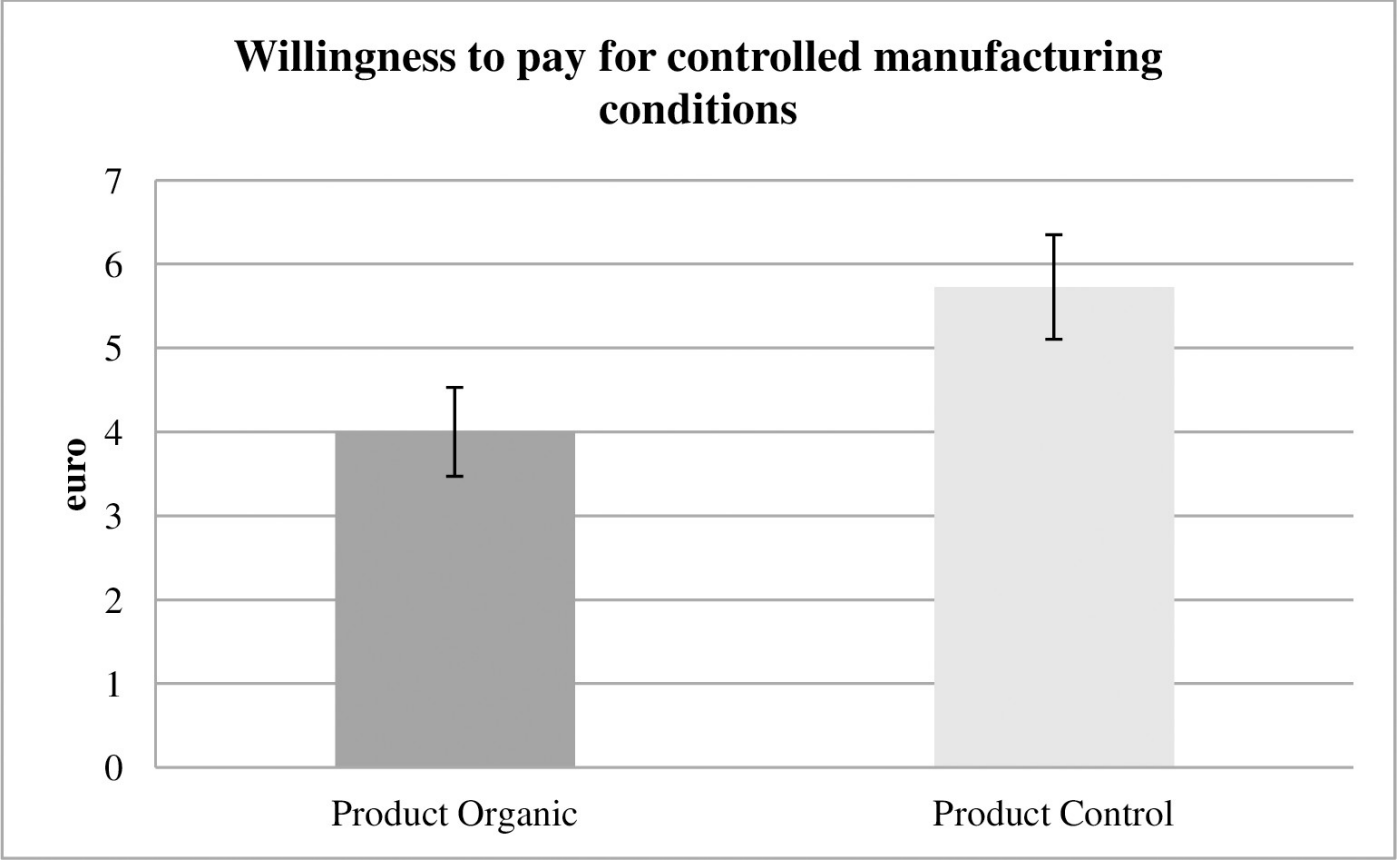
WTP product treatments. In Product Organic, subjects pay highly significantly less money to ensure controlled manufacturing standards compared with Product Control. Thus, static moral self-licensing takes place.

We hypothesized that static moral self-licensing takes place, that is, subjects treat orthogonal, ethically relevant aspects as if they were substitutes in product choice. Therefore, we expected that subjects in Product Organic would pay less money to ensure FWF-controlled manufacturing standards than in Product Control, as reflected in a lower switch point in the price list. The hypothesis was validated. In Product Control, the average switch point was 5.73 euro. We have seven multiple switchers in the sample. Our findings remain robust if we exclude them or use the first switch point, last switch-point, or median switch-point to approximate their willingness to pay. (See the robustness checks in Appendix A in S1 Appendix. for details.) In Product Organic, the average switch point and therefore WTP was 4.00 euro (Fig 2). This is a decrease of 30% (p = 0.002, one-sided t test), which is highly significant. We will speak of “highly significant” if the p value is below 0.01.

Subjects know from the instructions that sewing the towel is a very different production step than growing the cotton because the people involved and ecological consequences differ. Nevertheless, they attribute less value to guaranteeing minimum working standards when they know that their towel is made from organic cotton. Potentially, subjects already have a fairly good moral conscience in the latter case and accordingly care less about the workers in manufacturing. Thus, we find that static moral self-licensing exists.

### Beliefs about static moral self-licensing

**Fig 3:**
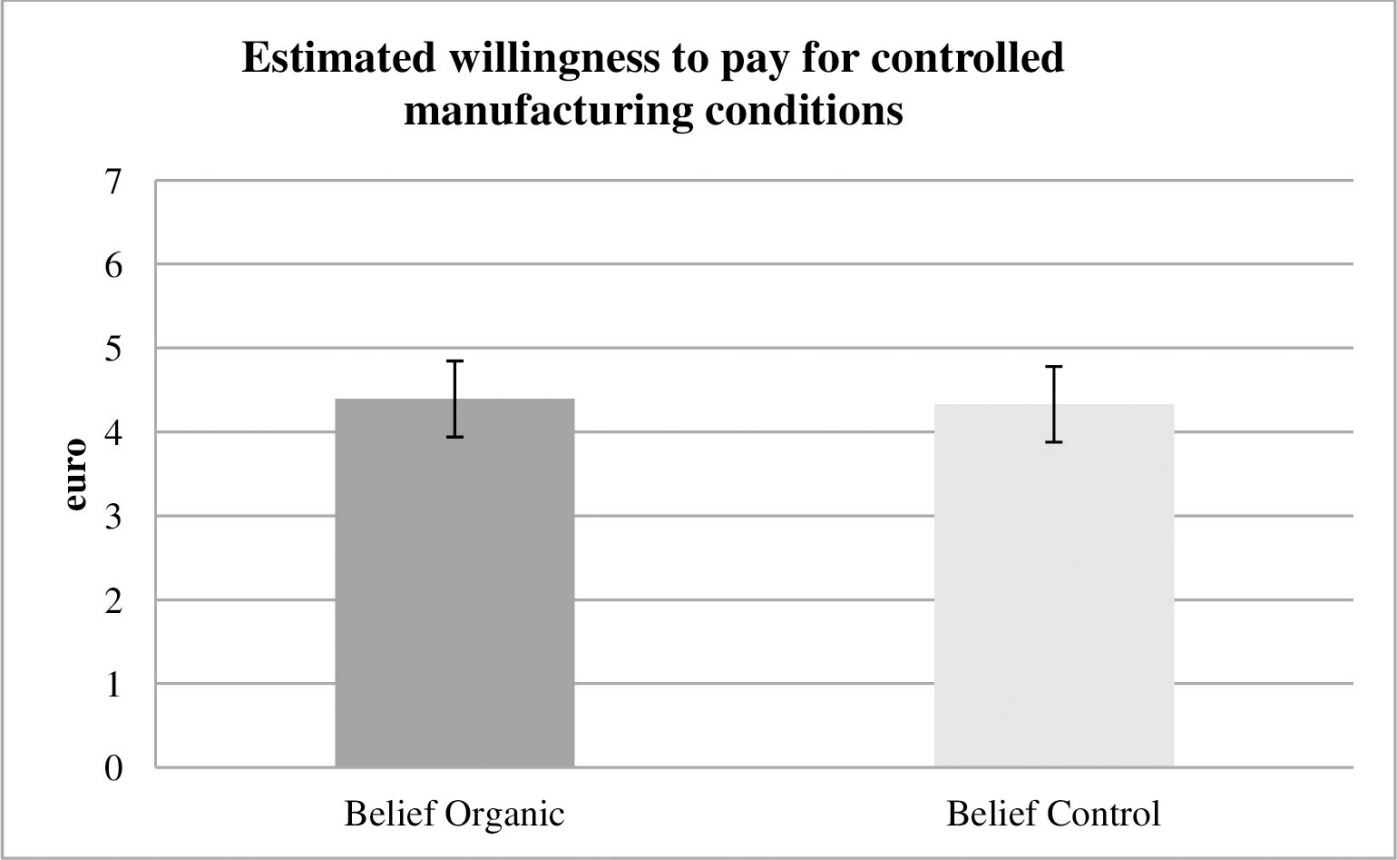
Estimated WTP belief treatments. In the Belief treatments, subjects guess the average WTP for controlled manufacturing in Product Organic, resp. Product Control. The data displays no significant difference in estimates at any conventional level. Thus, subjects in Belief Organic are not aware that static moral self-licensing may take place.

Do uninvolved people expect static moral self-licensing to take place? To consciously counter the effects of static moral self-licensing, people must be aware of it. We hypothesized, that for at least three reasons, this phenomenon may not be the case. Our data from Belief Organic demonstrates that subjects are not aware of the effects of static moral self-licensing (Fig 3). Estimates for switch points are at any conventional level and are virtually identical to those from Belief Control (4.39 vs. 4.33, p = 0.89, two-sided t test).

### Dynamic moral self-licensing

**Fig 4:**
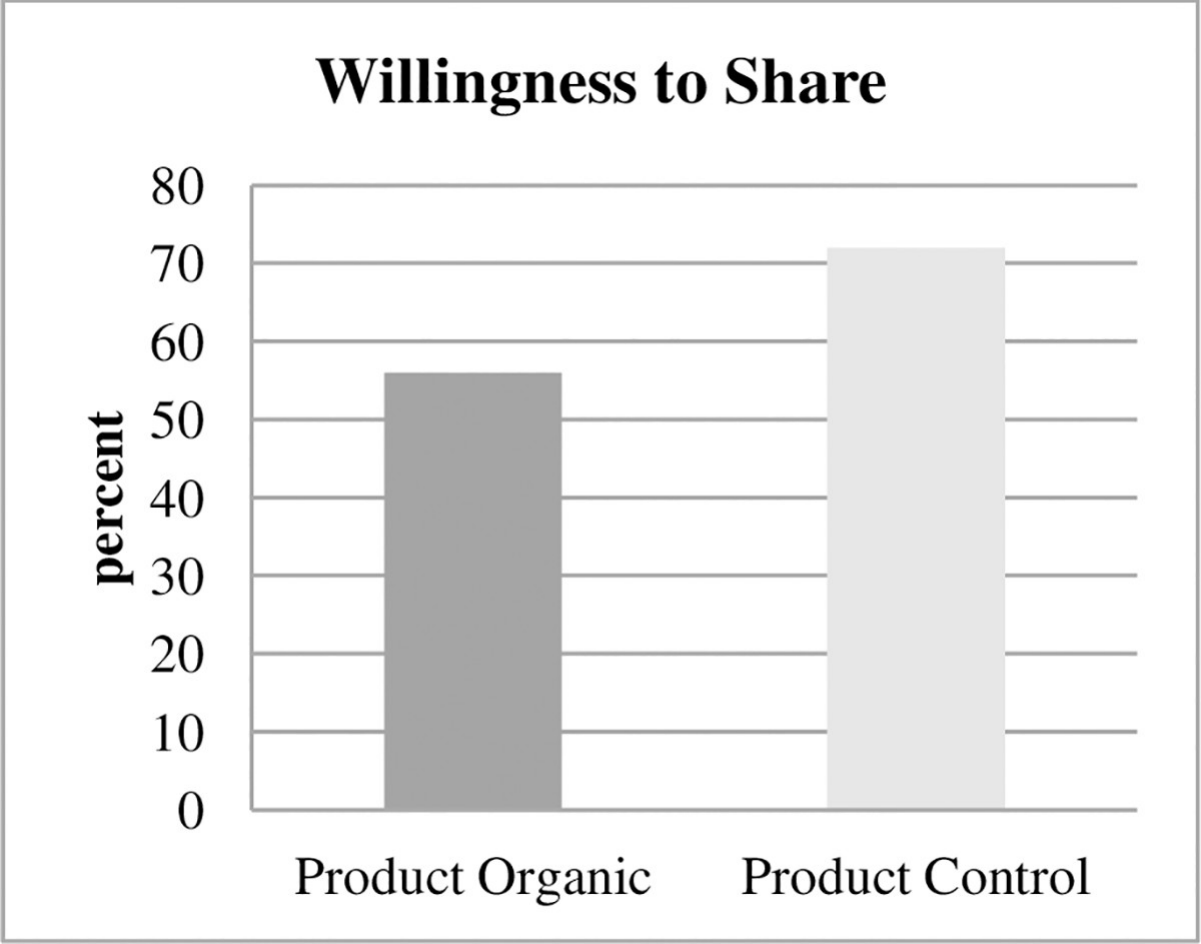
Willingness to share product treatments. Subjects in Product Organic are significantly less likely to share their show-up fee of euro with local refugees than subjects in Product Control.

At the end of the study, approximately half an hour later, subjects in the Product treatments were asked whether they wanted to share their show-up fee of 2 euros with refugees from a local refugee camp. Subjects did not know beforehand that they would be confronted with this decision. If static moral self-licensing has spillovers in time, subjects in Product Organic should less frequently share their show-up fee with refugees than subjects in Product Control, and this is what we observed (Fig 4): 72% of subjects are willing to share in Product Control, and 56% of subjects are willing to share in Product Organic (p = 0.048, one-sided test of proportions). As subjects in Product Organic could expect to leave with a higher income than subjects in Product Control because of the higher costs of organic textiles, they may be even more inclined to donate. The data display the opposite effect, and this demonstrates that the effects of dynamic moral self-licensing may be stronger than this potential income effect.

**Fig 5:**
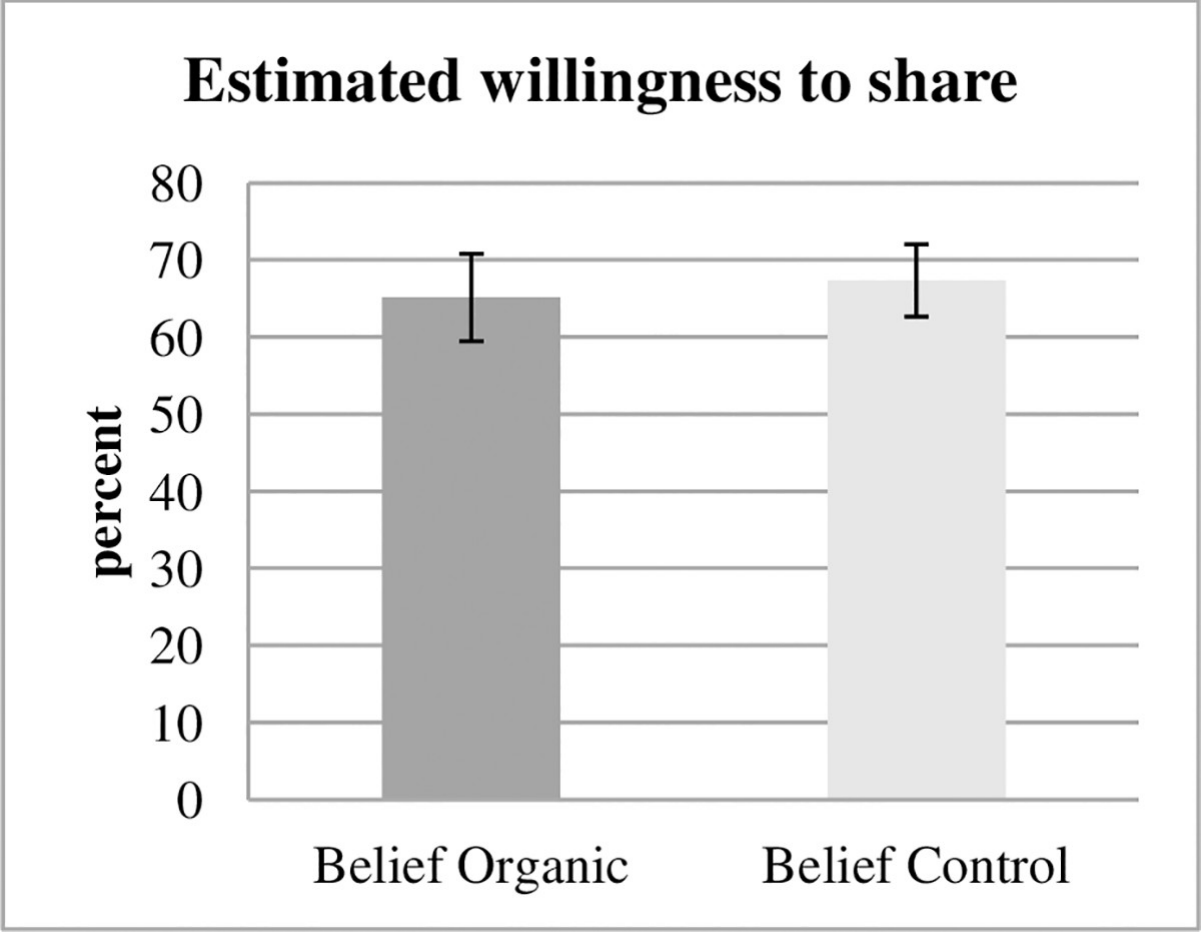
Estimated willingness to share belief treatments. Estimates from subjects in the Belief treatments for average willingness to share in the respective Product treatment.

Looking into the estimates from the two Belief treatments (Fig 5), there is again no significant difference in estimates of willingness to share with refugees for the two Product treatments (65% in Belief Organic vs. 67% in Belief Control, p = 0.68, two-sided t test). Outsiders therefore neither expect static nor dynamic moral self-licensing.

In summary, although the effects of moral self-licensing with regard to specific ethical dimensions are pronounced within decisions and over time, uninvolved parties do not anticipate their effects. This occurs even though uninvolved parties were monetarily incentivized and could earn much more money from making accurate guesses than from the flat payment they received for participating in the study. Many subjects’ beliefs were far from the correct answer such that they did not earn additional money for this task. 27% of subjects earned money for their estimates. Potentially, subjects in the Belief treatments merely indicate what they consider morally appropriate and do not figure in the effects of moral self-attribution. Subjects in Belief Control underestimated the willingness to pay in Product Control (p = 0.018, two-sided t test) but do not underestimate subjects’ willingness to donate to refugees.

## Discussion

In the following, we discuss our results. First, we demonstrate robustness by controlling for sociodemographic variables, personality traits, and attitudes toward refugees across Product treatments. Second, we discuss whether self-attribution is critical to our results. For this purpose, we run two new treatments that demonstrate that self-attribution seems to matter significantly. Third, we discuss the potential effects of decreasing attention in subjects and an alternative interpretation of why subjects pay less for certified working conditions in Product Organic. Fourth, we stress the need for follow-up research.

First, subjects were randomly assigned to treatments. This was also the case for the Product treatments. Nevertheless, a concern might be that treatment effects may be driven by differences in age or demographic background; reassuringly, Table 1 demonstrates that this is not the case. Subjects in Product Control and Product Organic do not significantly differ in age (p = 0.22, two-sided t test) or gender (p = 0.65, two-sided test of proportions). Furthermore, subjects have comparable amounts of money at their monthly disposal (p = 0.77, two-sided t test) and state no significant differences in their concern for their financial situation (p = 0.20, two-sided t test). In addition, subjects have similar opinions on whether Germany should support refugees fleeing war (p = 0.66, two-sided t test), political, religious or ethnic persecution (p = 0.65, two-sided t test), hunger, insufficient nourishment or natural disasters (p = 0.39, two-sided t test), or unemployment (p = 082, two-sided t test).

**Table 1:** Comparison of subjects in product control and product organic.

	Product Control	Product Organic	p value
Age †	21.82	21.06	0.22
Proportion of males ‡	76%	72%	0.65
Disposable income after rent (in euro) †	410	398	0.77
Financial concern (7-point Likert-scale) †	2.58	2.80	0.20
Machiavellianism †	56.88	58.06	0.47
Agreeableness †	12.9	12.4	0.37

The sociodemographics and the ethically relevant personality traits do not differ significantly between Product Control and Product Organic.

† two-sided t test, n = 100

‡ two-sided test of proportions, n = 100.

Regarding personality traits, we elicit measures that might influence ethical decision-making: Machiavellianism and agreeableness. Machiavellianism encompasses a variety of world views and actions characterized by a general disregard for other people to increase personal gain. Machiavellianism has been found to correlate negatively with ethical orientation and decision-making [54]. Machiavellianism has furthermore been positively linked to love for money [57] and economic opportunism[58]. Sacrificing money to improve the working conditions of unknown people without personal benefit would therefore not constitute Machiavellian behavior. The second trait of interest is agreeableness, and it is elicited in the Big Five. Agreeableness encompasses positive personal attitudes and behaviors, such as generosity, trustworthiness, and compassion [59, 60]. We find that subjects in Product Control and Product Organic show similar levels of Machiavellianism (p = 0.47, two-sided t test) and agreeableness (p = 0.37, two-sided t test). These similarities in sociodemographics and personality traits suggest that differences across Product treatments are based on other factors, that is, differences in decision contexts.

Second, a question could be whether self-attribution is critical for the treatment effect we observe in the Product treatments. An argument could be made for an alternative driver, that is, subjects' utility functions are concave regarding fulfilling separate ethical dimensions of products. This could imply a lower marginal utility in the organic treatment and lead to a lower WTP in Product Organic compared with Product Control. A related argument could be that subjects in Product Organic bunched the ethical attributes differently and therefore became less willing to pay for the certified sewing conditions (we thank an anonymous referee for pointing this out). If subjects in Product Organic consider the organic cotton and certified sewing conditions as part of one ethical factor, this could reduce their willingness to pay for the individual certificates, although they address very diffferent ethical aspects from a consequentialist viewpoint.

Thus, a question arises as to whether self-attribution significantly affected our results. Do people indeed need self-attribution to find some minimal yet plausible excuse to pay less in Product Organic? This question motivated us to run two additional treatments: Lottery Control and Lottery Organic. These treatments are similar to the original product choice treatments, respectively, with only one difference: much less room for self-attribution. The instructions stress that it is perfectly random regarding whether a subject decides about towels made from conventional or organic cotton. In this manner, subjects deciding about organic towels may attribute the fulfillment of this ethically relevant criterion less to themselves. Accordingly, fulfilling this criterion may not serve as a morally relevant excuse anymore, such that subjects may care more about the upgrade to the fair wear certificate and about donating to refugees. These two treatments were run 19 months after the original Product treatments with 50 subjects per treatment. Unfortunately, we had to exclude one subject (in Lottery Control) due to incorrect handling of the price list.

In **Lottery Control** and **Lottery Organic,** the randomness is made salient to subjects as follows. Blue and yellow cards are alternatingly handed to subjects. Next, all subjects within a session receive the same information on the chain of production of textile products, on FWF standards, and on organic cotton production (as in the original Product treatments). Subjects learn that a random coin toss and the color of their card determine whether they subsequently decide about towels made of conventional or organic cotton. Thus, the design renders the following salient: subjects are randomly distributed to one of the two treatments: Lottery Control or Lottery Organic. After the coin toss, subjects receive their respective price lists and instructions (see Appendix II for details). In contrast, in the original Product treatments, assignment to the specific treatment was random. This randomness was neither stressed nor visually demonstrated.

If self-attribution matters, we expect that both moral self-licensing effects should be less pronounced in the Lottery treatments. The following simple model describes the approach. We capture the self-attribution from fulfilling the ethically relevant cotton criterion in the Product Organic treatment through *α*. In Product Control, in which towels are never made from ethically produced cotton, we set *α* to 0. In the Lottery treatments, we assume that it is $\frac{\alpha }{2}$ because subjects know that fulfilling the organic cotton criterion is random and has a probability of 50%. In Product Organic, *α is* 1.

We assume that utility is separable between the moral and the monetary dimensions, and quasilinear in monetary payments. Utility from receiving a monetary amount *m* and having a moral value of *α* is thus given by *u*(*α*)+*m*. We assume that u is three times continuously differentiable. The monetary amount m_*_(*α*) that renders the agent indifferent between receiving money or receiving an additional moral value of *f* is given by m_*_(*α*) = *u*(*α*+*f*)−*u*(*α*)

Writing this as

$$m_{*}(\alpha )=u(\alpha +f)-u(\alpha )=\int_{0}^{f}\;u\prime (\alpha +t)dt,$$

we find that

$${m\prime }_{*}(\alpha )=\int_{0}^{f}u\prime \prime (\alpha +t)dt\;\mathrm{and}\;{m\prime \prime }_{*}(\alpha )=\int_{0}^{f}u\prime \prime \prime (\alpha +t)dt.$$

In particular, under risk aversion, *u*′′<0, m_*_ is decreasing in α. Moreover, if the agent is prudent in the sense of Kimball [61] in the moral dimension, *u*′′′>0, then m_*_ is convex. Conversely, imprudence, *u*′′′<0 implies the concavity of m_*_.

Thus, for a prudent agent, we have by Jensen’s inequality

$$m_{*}\left(\frac{\alpha }{2}\right)<\frac{1}{2}m_{*}(\alpha )+\frac{1}{2}m_{*}(0);$$

thus,

$$m_{*}\left(\frac{\alpha }{2}\right)-m_{*}(\alpha )<m_{*}(0)-m_{*}\left(\frac{\alpha }{2}\right),$$

that is, $m_{*}\left(\frac{\alpha }{2}\right)$ is closer to m_*_(*α*) than to m_*_(0). In the imprudent case, $m_{*}\left(\frac{\alpha }{2}\right)$ is closer to m_*_(0) than to m_*_(*α*).

In the Lottery treatments, self-attribution of the morally relevant organic cotton should amount to the same value $\frac{\alpha }{2}$. Therefore, the WTP for a second, morally relevant criterion should be comparable across the two Lottery treatments. Indeed, the data shows that WTP is statistically comparable.

**Fig 6:**
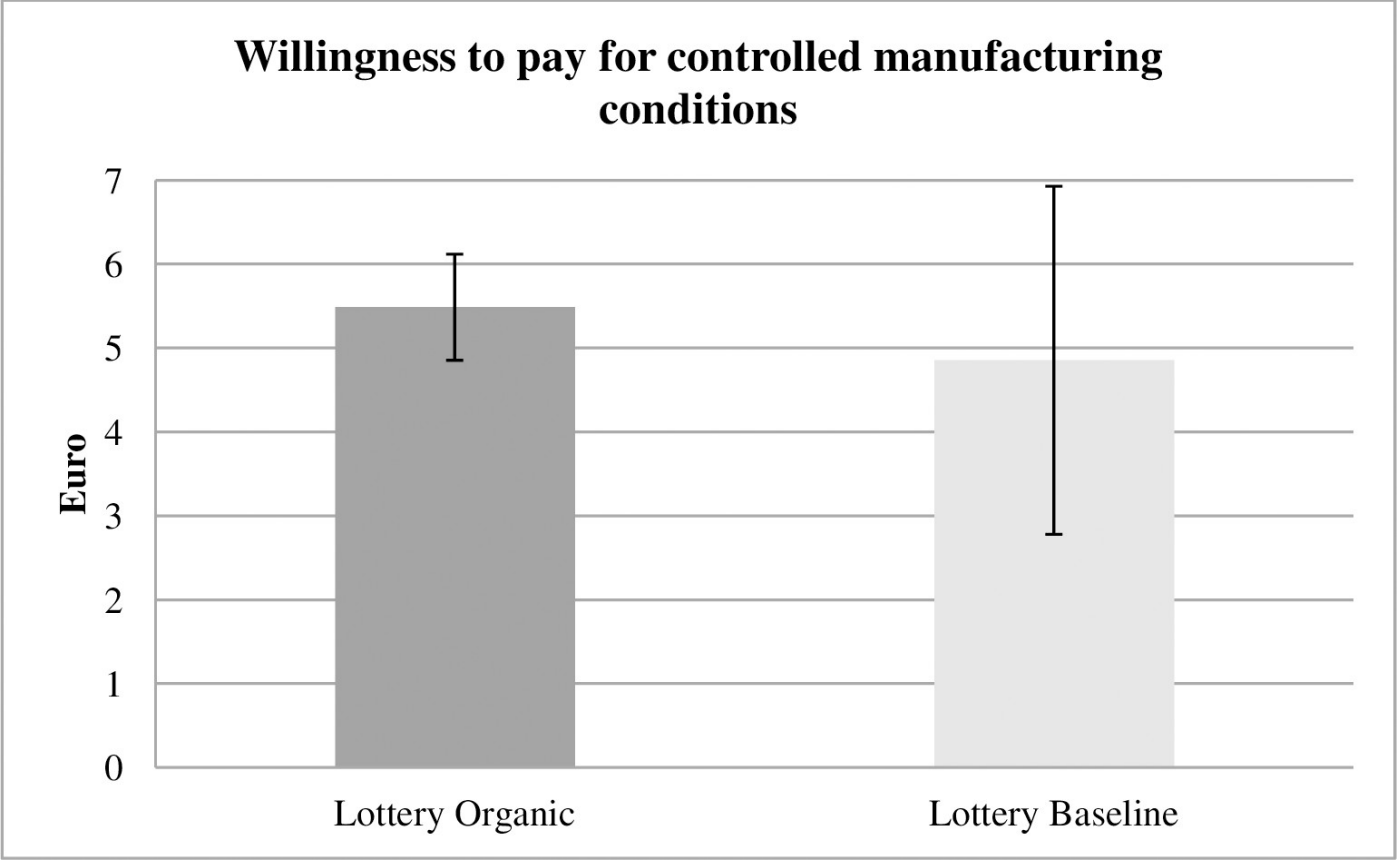
WTP lottery treatments. In the Lottery treatments, the static moral self-licensing effect disappears because there are no significant differences in the WTP for controlled manufacturing conditions. If anything, the pattern reverses based on a tendency.

In Lottery Organic, the average WTP for controlled manufacturing conditions is 5.49 euro, and it is 4.86 euro (p = 0.30, two-sided t test) in Lottery Control (Fig 6). As hypothesized, the moral self-licensing effect disappears.

**Fig 7:**
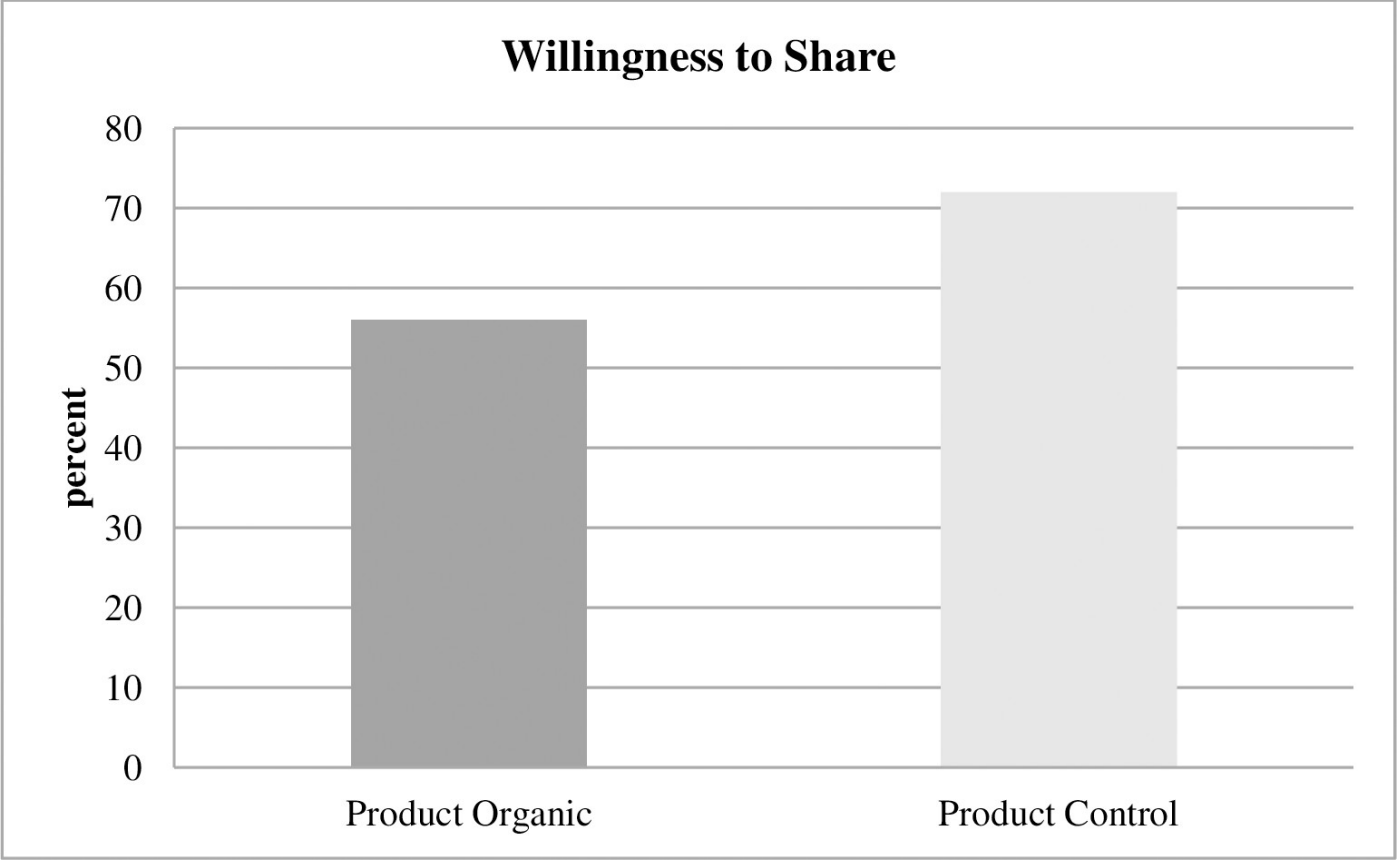
Willingness to share lottery treatments. Compared with the Product treatments, the share of donations is statistically comparable between subjects in the organic and the control treatment. The effects of dynamic moral self-licensing disappear in the Lottery treatments.

Analogously to the Product treatments, all subjects in the Lottery treatments were offered to share their show-up fee of 2 euro with local refugees. The share of subjects willing to donate is 66% in Lottery Organic and 62% in Lottery Control (Fig 7). In accordance with the hypothesis, the difference is not statistically significant (p = 0.68, two-sided test of proportions). The results suggest that moral self-attribution differs if the role of randomness in the experiment is emphasized.

A direct comparison to the original Product treatments, elicited 19 months earlier, must be used with some caution because, of course, ethical values in the population of interest may change over time. Notably, the general attitude toward refugees could have changed substantially between the sessions, which is why we refrain from conducting comparisons between the willingness to share between the Product and the Lottery treatments. Reassuringly though, when we ask our subjects whether Germany should support refugees fleeing from different causes, comparing the Product and the Lottery treatments, we find no significant differences. This is valid whether refugees are fleeing war (p = 0.96, two-sided t test), political, religious or ethnic persecution (p = 0.30, two-sided t test), hunger, insufficient nourishment or natural disasters (p = 0.37, two-sided t test), or unemployment (p = 0.21, two-sided t test).We find that compared with the original Product treatments, the average WTP for controlled manufacturing conditions does not change (p = 0.47, two-sided t test). When examining at the organic treatments only, however, subjects in Lottery Organic are willing to pay significantly more than subjects in Product Organic (5.49 euro vs. 4.00 euro, p = 0.01, two-sided t test). This finding is another indication that subjects care more about additional, ethically relevant facets if it is more difficult to attribute the fulfillment of the ethically relevant criterion regarding organic cotton to themselves.

Third, an argument could be that other drivers could play a critical role in the original Product treatments or that the effects in the Product treatments could be driven by decreasing attention when reading the instructions. Maybe, subjects no longer focus on what other ethical upgrades are possible for a towel once they learn of the first aspect (cotton production). Likewise, subjects may have not paid attention to the later donation decisions. However, as a driver of the differences across the Product treatments, this seems unlikely. Notably, instructions were identical in length across Product treatments. All subjects learned about cotton production first, then about the conditions in the sewing sector, and then (approximately half an hour later) about the donation opportunity.

Fourth, further research in different contexts is necessary to explore how general the moral self-licensing effects are that we documented in our study. Of course, as the first step, further research could also vary the design we used, that is, allow subjects to decide about certified cotton instead of the Fair Wear certification. Another important follow-up focus could be market analyses because maybe for firms, offering products that fulfill exactly one ethical criterion, for example, ecological cotton in textile items, is a smart strategy for selling products to customers with easy-to-ease ethical concerns. A further possibility is that leaving moral wiggle room (see Dana et al. [62], van der Weele [63], Bartling et al. [64], Grossman [65], Grossman and van der Weele [66], Freddi [67], Serra-Garcia and Szech [68], and Golman et al. [69] for a recent overview) and/or room for motivated reasoning [70, 71] could further decrease morally responsible behavior in customers. This potential interaction of moral self-licensing and other well-known mechanisms providing moral excuses is also a topic for further research.

## Conclusion

Our data documents that people behave as if there was strong substitutive relations between ethically relevant, non-correlated product dimensions. Our data thus suggest that static moral self-licensing exists. Fulfilling one ethical dimension in a product choice seems to ease moral conscience in a later, unrelated yet morally relevant context. Thus, different aspects of moral self-licensing and static (across morally relevant dimensions at one point in time) over different points in time seem to coexist.

Outsiders were not observed to infer that moral self-licensing could occur. Although monetarily incentivized to make accurate estimates, subjects were observed to be completely oblivious to effects of moral self-licensing, both static and dynamic. Therefore, it may have value for social and political debates on the potential effects of moral self-licensing. Firms know better than ordinary customers about effects of moral self-licensing and other mechanisms of moral excuses. Providing such excuses to customers may be a profitable approach. If so, political and societal debate should be aware of these mechanisms.

## Supporting information

S1 Appendix (DOCX)
