# Supplementary material for: A little good is good enough: Ethical consumption, cheap excuses, and moral self-licensing
Source: PLoS One. 2020 Jan 15;15(1):e0227036. doi: 10.1371/journal.pone.0227036 (PMC6961941; doi:10.1371/journal.pone.0227036)
Supplement: S1 Appendix — (DOCX) [file pone.0227036.s001.docx]

**Appendix**

**Robustness**

In the following, we study the robustness of our effects. First, we look in detail into the behavior of multi-switchers. Second, we control for income effects. Neither of them affects the results.

When eliciting the willingness to pay with the help of a price list, there is the possibility of subjects giving inconsistent answers, i.e., switching multiple times between the two product options. In order to ensure that those multi-switchers do not affect the results, we test for robustness using different measures of willingness to pay for multi-switchers. We therefore analyze the following specifications in the data for handling multi-switchers as well: excluding multi-switchers from the analysis, using the first switch-point, using the mean switch-point, using the median switch-point, and using the last switch-point (Table 2).

| **Switch-point without multi-switchers** | | | |
| --- | --- | --- | --- |
| Product Control | Product Organic | p value (one-sided) | n |
| 5.64 | 3.78 | 0.0015** | 93 |
| **First switch-point (including multi-switchers)** | | | |
| Product Control | Product Organic | p value (one-sided) | n |
| 5.61 | 3.70 | 0.0008*** | 100 |
| **Mean switch-point (including multi-switchers)** | | | |
| Product Control | Product Organic | p value (one-sided) | n |
| 5.73 | 4.00 | 0.0021*** | 100 |
| **Median switch-point (including multi-switchers)** | | | |
| Product Control | Product Organic | p value (one-sided) | n |
| 5.71 | 3.85 | 0.0011*** | 100 |
| **Last switch-point (including multi-switchers)** | | | |
| Product Control | Product Organic | p value (one-sided) | n |
| 5.89 | 4.29 | 0.0075*** | 100 |

**Table 2. Controlling for multi-switchers.**

Switch-points in the Product treatments, depending on different specifications for multi-switchers; p values presented are from the t tests between the treatments. Significance levels marked as follows: * p<0.1, ** p<0.05, *** p<0.01

The differences in willingness to pay between the treatments remain significant for all types of measurement. Thus, the effect is robust to different ways of addressing the case of multiple switchers.

Another important aspect regarding the robustness of the findings is the potential role of disposable income. Willingness to pay for controlled manufacturing conditions could be dependent on the financial background of subjects. As our sample is made up of students, maximizing the payoff from the experiment in order to get by could specifically matter for students with low disposable income. Reassuringly, we do not see any income effects, as there are no significant correlations between the willingness to pay for controlled manufacturing conditions and the monthly money at disposal (ρ=-0.09, p=0.35) or the subjects’ financial concern (ρ=-0.0203, p=0.84). We thus find that our results are robust, both to multi-switching and to financial background of subjects.

**Instructions**

**Instructions of the Product treatments**

*In the following, we present the instructions from Product Control and Product Organic. The instructions were presented on paper sheets, including a cover sheet and a prize list on the back of the cover sheet.*

**Welcome to this Study!**

Thank you for participating in an economic study at the Karlsruhe Institute of Technology (KIT).

As in all economic studies at KIT, all circumstances described in the following are true. Your decisions will be implemented exactly as described.

We would ask you to keep quiet during the study. If you have questions, please indicate by slightly opening your door. Your questions will then be answered at your cubicle.

**Here, please fill in your subject ID.**

**Subject-ID: __________________**

**Please start reading the two pages inside the cover sheet before attending to the list on the back page.**

**The production line of the garment industry**

In the course of this study, you will choose between two towels, which differ in their way of production.

En route from the cotton plant to the ﬁnal product ready to sell, a towel passes several production steps. Two essential production steps are the cultivation of cotton and the sewing of the ﬁnal product. These production steps are often done in regions far away from each other. This is why the conditions of the different production steps often vary widely.

Relating to the production standards in both production steps – cultivation and sewing - there exist established labels.

**What does “certified organic cotton” mean?**

This label is relevant to the cultivation of cotton. In contrast to conventionally grown cotton, certiﬁed organic cotton (organic cotton) is cultivated without chemically produced fertilizers, defoliants, or pesticides. Also the use of genetically modiﬁed organisms (GMOs) is prohibited. These conditions must be met in order to become certiﬁed and additionally, the soil must have been free from synthetic agrochemicals for three years. In the context of the certiﬁcation process, the plantations are audited at least once a year from independent institutions.

**What does “Fair Wear Foundation” mean?**

This label refers to the sewing of the ﬁnal product. The Fair Wear Foundation (FWF) is an international veriﬁcation initiative. The affiliated producers in the garment industry commit to the step-by-step implementation of social minimum standards in their manufacturing factories. This is audited by independent parties. Among the labor standards of the FWF are: employment is freely chosen, no discrimination in employment, no exploitation of child labor, freedom of association and the right to collective bargaining, payment of a living wage, no excessive working hours, safe and healthy working conditions, and legally binding employment relationships.

*Only subjects in Product Control received the following sheet.*

**Your task**

In this study, you will make a choice for a towel. You will receive in any case a towel made of conventional cotton of the size of 100cm x 50cm that will be handed out to you when the study has ended. All the towels in this study have the same grammage of 450g/m² and are held in neutral colors.

**Price list and remuneration**

On the back of the cover sheet is a list. In each row you will have the choice between Option A and Option B.

**Option A:** You will receive a towel made of conventional cotton without Fair Wear certification. Additionally, you will receive a monetary amount.

**Option B:** You will receive a towel made of conventional cotton with Fair Wear certification. You will not receive an additional monetary amount.

The monetary amount in Option A varies over the rows of the table. It starts at 12.00 euro and decreases in every row by 25 cent.

Please make a decision for every row by marking exactly one of the two options in every row.

Afterwards, one of the rows will be randomly selected. The probability is 1 out of 48 for every row. Your decision in the selected row will be exactly implemented as described. That means that you will receive your chosen towel and – depending on the decision – additional money.

Please note: If a row is chosen in which you did not mark exactly one option, you will not receive anything.

For your participation in the study, you will additionally receive 2 euro.

There is a colored piece of paper with your participation number attached to the cover sheet. This piece of paper serves as your coupon with which you can pick up your payout (towel and money) at the KD2 Lab as soon as you have received the respective email. Your remuneration will be bagged so that the person handing it out will not know the content.

Please keep the coupon until your remuneration. Otherwise, you cannot be remunerated.

Take your time to make yourself aware of the differences between the two options.

*Only subjects in Product Organic received the following sheet.*

**Your task**

In this study, you will make a choice for a towel. You will receive in any case a towel made of organic cotton of the size of 100cm x 50cm that will be handed out to you when the study has ended. All the towels in this study have the same grammage of 450g/m² and are held in neutral colors.

**Price list and remuneration**

On the back of the cover sheet is a list. In each row you will have the choice between Option A and Option B.

**Option A:** You will receive a towel made of organic cotton without Fair Wear certification. Additionally, you will receive a monetary amount.

**Option B:** You receive a towel made of organic cotton with Fair Wear certification. You will not receive an additional monetary amount.

The monetary amount in Option A varies over the rows of the table. It starts at 12.00 euro and decreases in every row by 25 cent.

Please make a decision for every row by marking exactly one of the two options in every row.

Afterwards, one of the rows will be randomly selected. The probability is 1 out of 48 for every row. Your decision in the selected row will be exactly implemented as described. That means that you will receive your chosen towel and – depending on the decision – additional money.

Please note: If a row is chosen in which you did not mark exactly one option, you will not receive anything.

For your participation in the study, you will additionally receive 2 euro.

There is a colored piece of paper with your participation number attached to the cover sheet. This piece of paper serves as your coupon with which you can pick up your payout (towel and money) at the KD2 Lab as soon as you have received the respective email. Your remuneration will be bagged so that the person handing it out will not know the content.

Please keep the coupon until your remuneration. Otherwise, you cannot be remunerated.

Take your time to make yourself aware of the differences between the two options.

*After the subjects made their choice and filled out the price list, they proceeded to fill out the personality and preference tests on a computer. These included the Big Five Inventory (BFI) [46] the Mach-IV test for Machiavellianism [47] as well as the Preference Survey Module [48], and further questions on social and political preferences.*

*After completing the questionnaires, subjects from Product Control and Product Organic could decide whether they wanted to share their show-up fee with local refugees. They did not know in advance to be confronted with this decision.*

**Now, you have the opportunity to share your show-up fee of 2 euro in a 1:1 ratio. You keep one half and the other half benefits local refugees.**

- Share the show-up fee (keep 1 euro, 1 euro for refugees)
- Don’t share the show-up fee (keep 2 euro, 0 euro for refugees)

**Instructions of the Belief treatments**

*In each Belief treatment, subjects were asked to guess the behavior of subjects from a Product treatment. Therefore, in addition to the instructions of the respective Product treatment (see Appendix II. a.), subjects in a Belief treatment also received the following, additional instructions. The additional instructions were identical for Belief Control and Belief Organic. The footnotes beneath the payout table were part of the instructions.*

**Welcome to this Study!**

Thank you for participating in an economic study at the Karlsruhe Institute of Technology (KIT).

As in all economic studies at KIT, all circumstances described in the following are true. Your decisions will be implemented exactly as described.

We would ask you to keep quiet during the study. If you have questions, please set the wheel on your door to red.

In this study, you will be shown the instructions of subjects of a previous study. These instructions are on the colored sheets.

Please read these instructions carefully. Later, you will be asked to estimate how the subjects of the previous study behaved. This determines a part of your remuneration.

In any case you will receive a show-up fee of 7 € for participating in this study.

**Please read the colored sheets first and take a look at the list. Then continue with the sheets on white paper that contain your instructions.**

**Your task**

You had the opportunity to read the instructions for previous subjects. In that study, subjects were asked for their willingness to pay^^[[1]](#footnote-1)^^ for fair manufacturing conditions with the aid of a price list. This willingness to pay could be located within the whole range of the price list.

**Please estimate the average willingness to pay among the previous subjects.^^[[2]](#footnote-2)^^**

Depending on the accuracy of your estimation, you can earn additional money. The closer you get to the average willingness to pay of the previous subjects, the more additional money you can earn.^^[[3]](#footnote-3)^^ This can be seen in detail in the following table.

**Payout table**

| **Deviation in cent** | **Additional money in euro** |
| --- | --- |
| 0 | 5.00 |
| 10 | 4.75 |
| 15 | 4.44 |
| 20 | 4.00 |
| 25 | 3.44 |
| 30 | 2.75 |
| 35 | 1.94 |
| 40 | 1.00 |
| 44 | 0.16 |
| 45 or more | 0 |

**Please indicate how high was the average willingness to pay of previous subjects (only values between 0.25 € and 12.25 € are technically permissible).**

**_________ Euro**

**Your Subject-ID: _________**

*After the subjects in the Belief treatments completed the personality and preference questionnaires (these were identical to the questionnaires in the Product treatments), they were asked to estimate what percentage of the previous subjects in the respective Product treatment were willing to donate to local refugees. Subjects did not know they would be confronted with this second estimation task beforehand.*

In the following, you can see a screenshot of another decision option of previous subjects.

-- Begin Screenshot --

**Now, you have the opportunity to share your show-up fee of 2 Euro in a 1:1 ratio. You keep one half and the other half benefits local refugees.**

- Share the show-up fee (keep 1 Euro, 1 Euro for refugees)
- Don’t share the show-up fee (keep 2 Euro, 0 Euro for refugees)

-- End Screenshot --

**Please estimate what percentage of the subjects was willing to share their show-up fee of 2 euro in a 1:1 ratio, thus picking the upper option.**

Depending on the accuracy of your estimation, you can earn additional money.*

| **Payout table** | |
| --- | --- |
| **Deviation in percentage points** | **Additional money in euro** |
| 0 | 5 |
| 1 | 4.8 |
| 2 | 4.2 |
| 3 | 3.2 |
| 4 | 1.8 |
| 5 | 0 |

**Please indicate what percentage of the previous subjects, in your opinion, was willing to share their show-up fee of 2 Euro in a 1:1 ratio. Please enter the percentage in whole numbers. Values between 0 and 100 percent are technically permissible.**

_____ Percent

* Your payout for this estimation amounts to **max {5 -** $\frac{\boldsymbol{1}}{\boldsymbol{5}}$ **(r - x)², 0} euro**, whereas x is your estimation and r is the percentage of previous subjects that were willing to share their show-up fee.

**Instructions of the Lottery treatments**

*In the following, we present the instructions from Lottery. When arriving at the reception in order to register to the experiment, the subjects were in turns given a yellow or a blue card with the comment that the card would become relevant during the course of the experiment. The instructions consisted of two parts: The first part was distributed to all subjects at the beginning of the experiment and consisted of a cover sheet and two more pages.*

**Welcome to this Study!**

Thank you for participating in an economic study at the Karlsruhe Institute of Technology (KIT).

As in all economic studies at KIT, all circumstances described in the following are true. Your decisions will be implemented exactly as described.

We would ask you to keep quiet during the study. If you have questions, please indicate by slightly opening your door. Your questions will then be answered at your cubicle.

**Please read the following two pages carefully.**

**The production line of the garment industry**

In the course of this study, you will choose between two towels, which differ in their way of production.

En route from the cotton plant to the ﬁnal product ready to sell, a towel passes several production steps. Two essential production steps are the cultivation of cotton and the sewing of the ﬁnal product. These production steps are often done in regions far away from each other. This is why the conditions of the different production steps often vary widely.

Relating to the production standards in both production steps – cultivation and sewing - there exist established labels.

**What does “certified organic cotton” mean?**

This label is relevant to the cultivation of cotton. In contrast to conventionally grown cotton, certiﬁed organic cotton (organic cotton) is cultivated without chemically produced fertilizers, defoliants, or pesticides. Also the use of genetically modiﬁed organisms (GMOs) is prohibited. These conditions must be met in order to become certiﬁed and additionally, the soil must have been free from synthetic agrochemicals for three years. In the context of the certiﬁcation process, the plantations are audited at least once a year from independent institutions.

**What does “Fair Wear Foundation” mean?**

This label refers to the sewing of the ﬁnal product. The Fair Wear Foundation (FWF) is an international veriﬁcation initiative. The affiliated producers in the garment industry commit to the step-by-step implementation of social minimum standards in their manufacturing factories. This is audited by independent parties. Among the labor standards of the FWF are: employment is freely chosen, no discrimination in employment, no exploitation of child labor, freedom of association and the right to collective bargaining, payment of a living wage, no excessive working hours, safe and healthy working conditions, and legally binding employment relationships.

**Color of the card and decision**

Randomness will determine whether you decide between towels made of organic cotton or of conventional cotton.

During the registration you randomly received a blue or a yellow card. After all participants have made themselves familiar with the instructions, the experimenter tosses a coin.

If the coin shows heads, participants with a yellow card will decide over towels made of conventionally grown cotton. Correspondingly, participants with a blue card will decide over towels made of organically grown cotton.

If the coin shows tails, participants with a yellow card will decide over towels made of organically grown cotton. Correspondingly, participants with a blue card will decide over towels made of conventionally grown cotton.

**You will soon be informed about the result of the coin toss by a room announcement.**

*After all subjects had read their instructions, we made the following announcement:*

*1. “Dear participants, the coin will be tossed now.”*

*2. We tossed the coin. The coin toss was audible over the loudspeakers.*

*3. The coin shows heads (tails), I repeat: The coin shows heads (tails).*

*4. We will distribute further instructions. Please remain seated.*

*Subjects that could decide over a towel made of conventional cotton were handed out the following instructions after the coin toss and the announcement.*

**Your task**

In this study, you will make a choice for a towel. You will receive in any case a towel of the size of 100cm x 50cm that will be handed out to you when the study has ended. By chance you decide due to the color of your card on towels made of conventionally grown cotton. All the towels in this study have the same grammage of 450g/m² and are held in neutral colors.

**Price list and remuneration**

On the backs of the cover sheet is a list. In each row you will have the choice between Option A and Option B.

**Option A:** You will receive a towel made of organic cotton without Fair Wear certification. Additionally, you receive a monetary amount.

**Option B:** You will receive a towel made of organic cotton with Fair Wear certification. You do not receive an additional monetary amount.

The monetary amount in Option A varies over the rows of the table. It starts at 12.00 euro and decreases in every row by 25 cent.

Please make a decision for every row by marking exactly one of the two options in every row.

Afterwards, one of the rows will be randomly selected. The probability is 1 out of 48 for every row. Your decision in the selected row will be exactly implemented as described. That means that you will receive your chosen towel and – depending on the decision – additional money.

Please note: If a row is chosen in which you did not mark exactly one option, you will not receive anything.

For your participation in the study, you will additionally receive 2 euro.

There is a colored piece of paper with your participation number attached to the cover sheet. This piece of paper serves as your coupon with which you can pick up your payout (towel and money) at the KD2 Lab as soon as you have received the respective email. Your remuneration will be bagged so that the person handing it out will not know the content.

Please keep the coupon until your remuneration. Otherwise, you cannot be remunerated.

Take your time to make yourself aware of the differences between the two options.

*Subjects that could decide over a towel made of organic cotton were handed out the following instructions after the coin toss and the announcement.*

**Your task**

In this study, you will make a choice for a towel. You will receive in any case a towel of the size of 100cm x 50cm that will be handed out to you when the study has ended. By chance you decide due to the color of your card over towels made of organically grown cotton. All the towels in this study have the same grammage of 450g/m² and are held in neutral colors.

**Price list and remuneration**

On the backside of the cover sheet is a list. In each row you will have the choice between Option A and Option B.

**Option A:** You receive a towel made of organic cotton without Fair Wear certification. Additionally, you receive a monetary amount.

**Option B:** You receive a towel made of organic cotton with Fair Wear certification. You do not receive an additional monetary amount.

The monetary amount in Option A varies over the rows of the table. It starts at 12.00 euro and decreases in every row by 25 cent.

Please make a decision for every row by marking exactly one of the two options in every row.

Afterwards, one of the rows will be randomly selected. The probability is 1 out of 48 for every row. Your decision in the selected row will be exactly implemented as described. That means that you will receive your chosen towel and – depending on the decision – additional money.

Please note: If a row is chosen in which you did not mark exactly one option, you will not receive anything.

For your participation in the study, you will additionally receive 2 euro.

There is a colored piece of paper with your participation number attached to the cover sheet. This piece of paper serves as your coupon with which you can pick up your payout (towel and money) at the KD2 Lab as soon as you have received the respective email. Your remuneration will be bagged so that the person handing it out will not know the content.

Please keep the coupon until your remuneration. Otherwise, you cannot be remunerated.

Take your time to make yourself aware of the differences between the two options.

**Recruitment Process and Sample**

Recruitment for the experiment sessions began in November 2015 for the Product treatments, in April 2016 for the Belief treatments, and in June 2018 for the Lottery treatments. We excluded potential participants with more than 10 previous participations in experiments or two or more failures to appear. All participants were recruited from the pool of potentially willing participants via ORSEE (the sessions in 2015 and 2016) and Hroot (the sessions in 2018) [49, 50].

The majority of the sample are students of the KIT and neighboring colleges. The participation in the study was completely voluntary. In that sense our sample might not be a representative for the general population of students. No minors participated in the study. Table 3 gives an overview of the sociodemographics of the subjects.

| Total number of observations | 299 |
| --- | --- |
| Female | 99 |
| Male | 200 |
| Students | 287 |
| Unemployed | 6 |
| Employed or self-employed | 6 |
| Age range | 18 - 44 |
| Mean age | 22.2 |
| Median age | 21 |

**Table 3. Sociodemographics of our subjects.**

The vast majority of participants are students in their early twenties with a male to female ratio of 2:1.

In the following, we list the general rules for the recruitment pool as well as the contractor's declaration that has to be signed when participating in an experiment. The participant rules for ORSEE and hroot remained the same.

***Participant rules***

*In order to participate in experiments carried out at the KD2Lab, you must register online. The registration is a declaration of your intent to participate in experiments. Multiple registrations in the system are not allowed. Participation in experiments is possible from the age of 18.*

*Before participating in an experiment you will receive a participation contract from the experimenter which you confirm with your signature before the start of the experiment.*

*The following rules apply to participation in the experiments:*

*For each experiment a certain number of persons registered here will receive an invitation by e-mail. Only persons invited via this platform are entitled to participate in the experiment.*

*After receiving an invitation by e-mail for a certain experiment, the participant must reply positively in order to be registered for this experiment. This reply is a binding promise to participate in this experiment.*

*For each experiment more participants will be invited than needed. Those who register first will take part in the experiment. Participants who have been invited and are present in time but cannot participate for reasons for which the experimenter is responsible, will be compensated for their attendance.*

*Participants are usually paid in cash. The amount paid depends typically on the participants' own decisions or on the decisions of the other experimental participants.*

*Punctual appearance of the participants is absolutely necessary. Each participant must arrive at least 15 minutes before the planned start of the experiment in front of the laboratory named in the invitation letter.*

*Participants should always schedule at least as much time for the experiment as stated in the invitation letter. However, the experimenters reserve the right to finish an experiment before the time has elapsed.*

*Participants who have declared their willingness to participate in a certain experiment and who do not appear or appear too late endanger the execution of the experiment due to a lack of participants. This behavior leads to a negative evaluation in the participant's profile. The experimenters reserve the right not to invite participants with too many negative evaluations to experiments.*

*If a participant has registered bindingly for an experiment, the participation can only be cancelled by giving an important reason up to 24 hours before the beginning of the experiment.*

*In some experiments webcam, audio or neurophysiological data (such as eye tracking or pulse) are collected. Whether such data is collected is deliberately not always communicated in the invitation.*

*By registering for participation in economic experiments, you agree to the above rules.*

***Data protection***

*Participant database*

*The data collected in the so-called "Recruitment System" (HROOT) of the KD2Lab are used exclusively for the organization of scientific experiments. This data will not be passed on to third parties. We use the data for the following purposes:*

- *to inform the participants about new laboratory or internet experiments and to invite them to them*
- *to check the appearance or non-appearance of the registered participants during experiments*
- *to make a scientifically motivated selection of participants for certain experiments (e.g. by gender, number of experiments already taken part or number of dates on which you did not appear)*

*Mandatory entries such as e-mail address and gender are a prerequisite for inviting you to our experiments and, if there is a scientific need, for filtering by gender. Filtering by gender can be crucial if, for example, researchers need a balanced ratio of women and men for their study.*

*Deletion from the participant pool*

*You have the right to have your data permanently deleted from the "Recruitment System". To do this, send an e-mail to the team address: team@kd2lab.kit.edu with the note "Delete". We will then delete all your personal data.*

*Alternatively, you can have your person assigned the status "inactive". If you would like to participate in experiments again at a later date. In this case we will switch your dataset back to the status "active" on your instructions.*

*Experiments*

- *During the execution of experiments, data is generated by the decisions to be made by the participants.*
- *This data is evaluated scientifically. The decision data is anonymized and cannot be assigned to any person. In this sense, participation in the experiments is anonymous.*
- *The generated, anonymized data will be used for scientific research papers and presentations which will be published.*
- *Experimental data will not be associated with the data in HROOT. There is no connection between the data generated in the experiment and the data in the “recruitment system”. Data collection of the participant’s name in the experiment is permitted only to create payout lists and must be deleted after that.*

*With participation in this experiment and signature of the participant agreement, you accept the anonymized use of experimental data for the research purposes of our scientists.*

*Do you agree to the rules and data security provision?*

***Contractor's declaration***

*I make the following declaration for the determination and payment of my fees.*

*I am aware*

1. *that the information contained in this declaration forms the basis for the determination of my fees*
2. *that I am obligated to notify any change to the information contained in this statement in writing without delay and that I have to pay back amounts which are incorrect due to incorrect or due to omitted, late or erroneous notification, in case too much has been paid.*
3. *that I have to pay any taxes to the relevant tax office myself,*
4. *that, to the extent that employment in the public service exists at the same time, I have to report as a sideline.*

*Hereby I commit myself to participate in the experiment presented by the project management. I accept the rules of the procedure and the payout mode, as they were made known and explained to me by the project management.*

**References (Appendix)**

[46] Rammstedt B, John OP. Kurzversion des Big Five inventory (BFI-K) Diagnostica 2005;51(4): 195–206.

[47] Christie R, Geis F. Studies in Machiavellianism (Academic Press, New York; 1970).

[48] Falk A, Becker A, Dohmen TJ, Huffman D, Sunde U. The preference survey module: A validated instrument for measuring risk, time, and social preferences. Working paper. SSRN Electronic Journal. 2016.

[49] Greiner B. Subject pool recruitment procedures: organizing experiments with ORSEE. Journal of the Economic Science Association 2015;1(1): 114-125.

[50] Bock O, Baetge I, Nicklisch A. hroot – Hamburg registration and organization online tool. European Economic Review 2014;71:117-120

1. The willingness to pay is the amount of money for which the subjects of the previous study switched from the right column (Fair Wear without additional money) to the left column (no Fair Wear, additional money). [↑](#footnote-ref-1)
2. The arithmetic mean of the willingness to pay of every subject is calculated. [If a subject has switched multiple times between the columns, we use the arithmetic mean of every willingness to pay. If a subject always preferred a towel without a Fair Wear certification, we define his willingness to pay as 0.25 €. If a subject always preferred the Fair Wear towel, we define his willingness to pay as 12.25 €.] [↑](#footnote-ref-2)
3. Your payout for this estimation amounts to **max {500 -** $\frac{\boldsymbol{1}}{\boldsymbol{4}}$ **(r – x)², 0} cent**, whereas x is your estimation and r the mean willingness to pay. [↑](#footnote-ref-3)
